# IFN-β Inhibits the Increased Expression of IL-9 during Experimental Autoimmune Uveoretinitis

**DOI:** 10.1371/journal.pone.0048566

**Published:** 2012-10-30

**Authors:** Yan Yang, Liping Du, Min Sun, Aize Kijlstra, Peizeng Yang

**Affiliations:** 1 Zhongshan Ophthalmic Center, Sun Yat-sen University, Guangzhou, P. R. China; 2 The First Affiliated Hospital of Chongqing Medical University, Chongqing Key Laboratory of Ophthalmology and Chongqing Eye Institute, Chongqing, P. R. China; 3 Eye Research Institute Maastricht, Department of Ophthalmology, University Hospital Maastricht, Maastricht, The Netherlands; Université Paris Descartes, France

## Abstract

**Purpose:**

It has been shown that IL-9 plays a proinflammatory role in the pathogenesis of certain autoimmune diseases. This study was designed to investigate the possible role of IL-9 in the development of experimental autoimmune uveoretinitis (EAU) and the effect of IFN-β on its expression.

**Methods:**

EAU was induced in B10RIII mice by immunization with interphotoreceptor retinoid-binding protein peptide 161–180 (IRBP_161–180_). IFN-β was administered subcutaneously to IRBP_161–180_ immunized mice every other day from day one before immunization to the end of the study. Splenocytes and draining lymph node (DLN) cells from EAU mice or control mice or EAU mice treated with IFN-β or PBS were stimulated with anti-CD3/CD28 or IRBP_161–180_ for 3 days. Naïve T cells cultured under Th1 or Th17 polarizing conditions were incubated in the presence or absence of IFN-β for 4 days. Effector/memory T cells were activated by anti-CD3/CD28 in the presence or absence of IFN-β for 3 days. IFN-β-treated monocytes were cocultured with naïve T cells or effector/memory T cells for 3 days. Culture supernatants were collected and IL-9 was detected by ELISA.

**Results:**

IL-9 expression in splenocytes and DLN cells was increased in EAU mice during the inflammatory phase and returned back to lower levels during the recovery phase. IFN-β in vivo treatment significantly inhibited EAU activity in association with a down-regulated expression of IL-9. In vitro polarized Th1 and Th17 cells both secreted IL-9 and the addition of IFN-β suppressed production of IL-9 by both Th subsets. Beside its effect on polarized Th cells, IFN-β also suppressed the secretion of IL-9 by effector/memory T cells. However, IFN-β-treated monocytes had no effect on the production of IL-9 when cocultured with naïve or effector/memory T cells.

**Conclusion:**

IL-9 expression is increased during EAU which could be suppressed by IFN-β.

## Introduction

Experimental autoimmune uveoretinitis (EAU) serves as an animal model for human uveitis and is widely used to dissect the immunopathological mechanisms in uveitis and to develop preventive or therapeutic strategies [1]. EAU can be induced by immunization with uveitogenic retinal antigens, such as retinal S antigen [2], interphotoreceptor retinal-binding protein (IRBP) [3]–[4] or their polypeptides, or by the adoptive transfer of uveitogenic T cells [5]–[6], suggesting that uveitis is a T cell-mediated, organ-specific autoimmune disease. IFN-γ-producing Th1 cells and IL-17-producing Th17 cells are two types of inflammatory T cells that play important roles in the development of EAU [7]–[9].

Recently, a new effector T cell subset, Th9 cells, has been identified [10]–[11], but its role in the pathogenesis of uveitis is not yet clear. Driven by the combined effects of TGF-β and IL-4, Th9 cells produce large amounts of IL-9 and IL-10. However, IL-9 is not only produced by Th9 cells. IL-9 was primarily described in mice and humans as a Th2 cytokine [12], but recent studies have identified it as a dominant cytokine produced by Th17 cells [13]–[14]. IL-9 has been shown to mediate Th17 cell differentiation [13], [15], and in an animal model, IL-9 neutralization and IL-9 receptor deficiency attenuates experimental autoimmune encephalitis [14]–[15]. These findings suggest that IL-9 may play a proinflammatory role during the development of autoimmune disease.

Previously, we showed that IFN-β could relieve EAU activity partially by down-regulating IFN-γ, IL-17 and up-regulating IL-10 production [16]. Whether IL-9 pays a role during the development of EAU has not yet been studied and whether IFN-β can modulate its production in this model is unclear and was therefore the subject of the study presented here. IL-9 expression by peripheral lymphocytes from spleen and draining lymph nodes was investigated in EAU mice, and the role of IFN-β in the production of IL-9 was tested both in vivo and in vitro. The results showed that IL-9 expression was increased in the active phase of EAU and that IFN-β was able to suppress the production of IL-9 both in vivo and in vitro.

## Methods

### Animals and Reagents

B10RIII mice (6–8 weeks), purchased from Jackson Laboratory, were housed in the animal facilities of the Chongqing Medical University. Cells were cultured in RPMI 1640 (Gibco BRL; Invitrogen) supplemented with 1% penicillin/streptomycin and 10% fetal bovine serum. IRBP_161–180_ (SGIPYIISYLHPGNTILHVD) was synthesized by Shanghai Sangon Biological Engineering Technology & Services Limited Company. CFA containing 1.0 mg/ml of Mycobacterium tuberculosis was obtained from Sigma-Aldrich (St. Louis, MO). Murine IL-12, IL-6, IL-23, and human TGF-β1 were purchased from R&D systems (Minneapolis, Minn, USA). Murine IFN-β was obtained from PBL (Piscataway, NJ, USA). Anti-CD3 and anti-CD28 antibodies were purchased from eBioscience (San Diego, CA, USA).

### Ethics Statement

All animals were treated according to the ARVO Statement for the Use of Animals in Ophthalmic and Vision Research. The protocol was approved by the Ethics Committee of the First Affiliated Hospital of Chongqing Medical University, Chongqing, China. (Permit Number: 2009-201009).

### EAU Induction and Treatment

B10RIII mice (8–12 weeks) were immunized with a 200 µl emulsion containing 50 µg IRBP_161–180_ in CFA subcutaneously, divided among the base of the tail and both thighs. Mice received an emulsion of 50 µl PBS and 150 µl CFA in the same way as a control. The clinical and histological scoring of EAU was performed according to criteria described previously [17]–[18]. Briefly, the severity of EAU was evaluated in a masked fashion on a scale of 0–4 based on the number, type, and size of lesions.

Murine IFN-β (1000 U in 100 ul PBS) or the vehicle PBS were administered subcutaneously to IRBP_161–180_ immunized mice every other day from day one before immunization until the end of the study.

### Cell Purification

CD4^+^CD62L^+/−^ T cells from normal mice and CD11b^+^ monocytes from the immunized mice on day 13 were purified from splenic single-cell suspensions by magnetic-activated cell sorting using a CD4^+^CD62L^+^ T cell and CD11b^+^ monocytes isolation kit respectively (Miltenyi Biotec, Palo Alto, CA). The purity of isolated cells was more than 95%, as identified by flow cytomety.

### Cell Culture

On day 13 or day 28 after immunization, splenocytes and draining lymph node (DLN; inguinal and iliac) cells (2×10^6^/ml) were stimulated with soluble anti-CD3/CD28 (1 µg/ml) or IRBP_161–180_ (20 µg/ml) for 3 days and the supernatants were collected to examine IL-9, IL-17 and IFN- γ expression.

CD4^+^CD62L^+^ naïve T cells (1.5×10^6^/ml) were stimulated with soluble anti-CD3/CD28 (1 µg/ml) for 4 days under Th1-polarizing (10 ng/ml IL-12) or Th17-polarizing (20 ng/ml IL-6, 5 ng/ml TGF-β1, and 10 ng/ml IL-23) conditions in the presence or absence of IFN-β (250 U/ml). CD4^+^CD62L^−^ effector/memory T cells (1.5×10^6^/ml) were stimulated with soluble anti-CD3/CD28 (1 µg/ml) for 3 days in the presence or absence of IFN-β (250 U/ml). Supernatants of the cell cultures were collected after centrifugation and used to detect the expression of IL-9.

### Coculture of IFN-β Treated Monocytes with CD4^+^CD62L^+/−^ T Cells

Monocytes were stimulated with or without IFN-β (250 U/ml) for 24 hours. After washing with PBS, they were cultured with CD4^+^CD62L^+^ naïve T cells or CD4^+^CD62L^−^ effector/memory T cells at a ratio of 1∶5 in the presence of soluble anti-CD3/CD28 (1 µg/ml) for 3 days. Supernatants were collected to detect the expression of IL-9.

### ELISA

Levels of IL-9 were measured using commercially available ELISA kits (eBioscience) according to the manufacturer’s instructions with a detection limit of 30 pg/ml. The expression of IL-17 and IFN-γ in cell culture supernatants was detected using Duoset ELISA development kits (R&D Systems, Minneapolis, MN) with detection limits of 15.6 pg/ml and 31.3 pg/ml, respectively.

### Statistical Analysis

Statistical significance was analyzed by Student’s *t* test and the Mann-Whitney U test. Data were expressed as the mean ± standard deviation (SD). *P*<0.05 was considered significantly different.

## Results

### IL-9 Expression is Increased during the Active Phase of EAU

**Figure 1:**
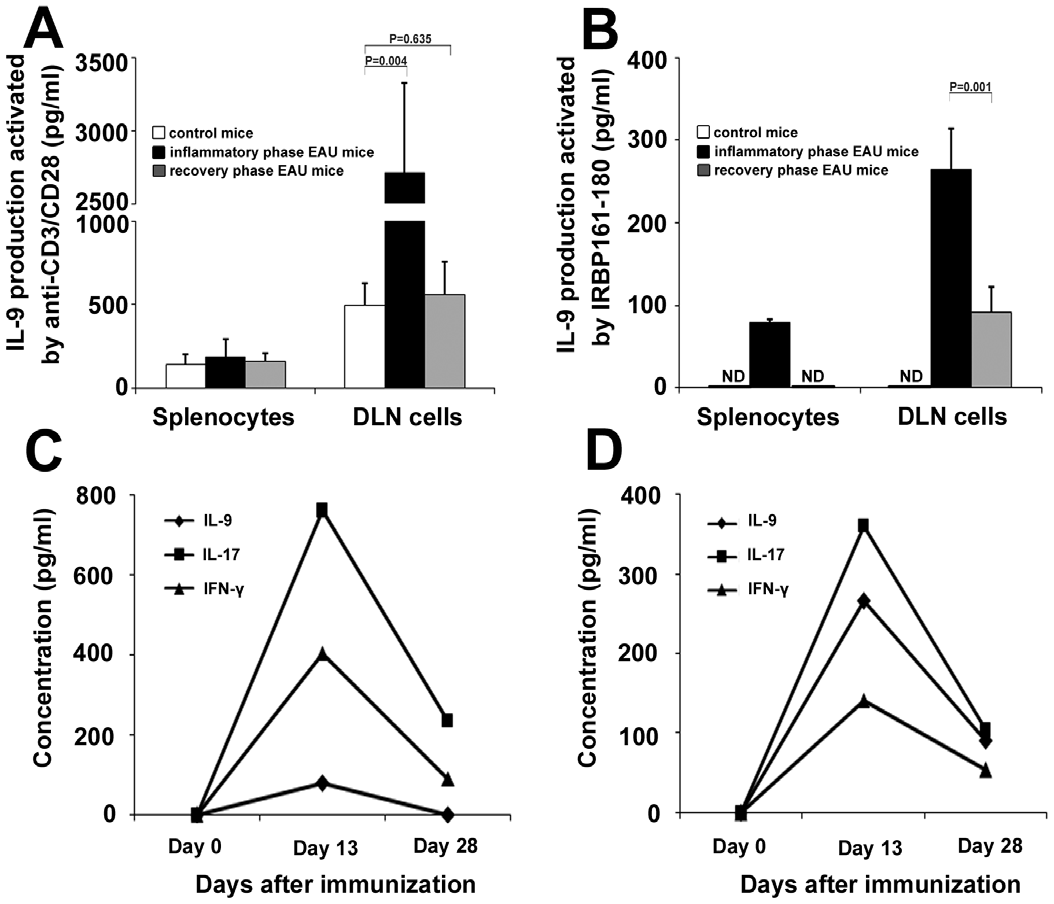
The expression of IL-9 in the EAU mice and the control mice. Splenocytes and DLN cells, obtained from the EAU mice (inflammatory phase and recovery phase) or control mice (n = 5 per group), were activated with anti-CD3/D28 (1 µg/ml) (A) or IRBP_161–180_ (20 µg/ml) (B) for 3 days. IL-9 was analyzed by ELISA. Splenocytes (C) and DLN cells (D), obtained from the immunized mice on indicated time points, were stimulated with IRBP_161–180_ (20 µg/ml) for 3 days, and the supernatants were collected for measuring IL-9, IL-17 and IFN-γ. Data are representative of three independent experiments. ND: not detected.

Splenocytes and DLN cells obtained from inflammatory phase EAU mice (day 13), recovery phase EAU mice (day 28), and control mice were stimulated with anti-CD3/CD28 or IRBP_161–180_ and the supernatants were assessed for IL-9 expression (Figure 1). The results showed that when activated by anti-CD3/CD28, IL-9 expression in DLN cells was significantly higher in the inflammatory phase EAU mice than the control mice (2712.6±616.7 pg/ml vs. 501.8±129.2 pg/ml, P = 0.004). During the recovery phase, the level of IL-9 decreased, and there was no difference as compared to levels observed in cells obtained from control mice (561.3±199.7 pg/ml vs. 501.8±129.2 pg/ml, P = 0.635). No differences were observed between inflammatory phase EAU mice, recovery EAU mice and control mice concerning the production of IL-9 (188.5±106.4 pg/ml, 163.7±47.8 pg/ml, 147.4±60.0 pg/ml, P = 0.357) when we tested splenocytes. Levels of IL-9 following anti-CD3/CD28 stimulation were much lower in splenocytes than in DLN cells.

When activated by the specific antigen IRBP_161–180_, IL-9 was detected in both splenocytes and DLN cells during the inflammatory phase of EAU. Much higher IL-9 was found in DLN as compared to splenocytes. In the recovery phase, IL-9 in splenocytes became undetectable. The production of IL-9 in IRBP stimulated DLN cells also decreased during the recovery phase as compared to the inflammatory phase but could still be detected (90.5±31.2 pg/ml vs. 265.1±49.0 pg/ml, P = 0.001). We also found that IRBP stimulated the expression of IL-9, IL-17 and IFN-γ and that these lymphokines reached their peak during the inflammatory phage of EAU (Figure 1C). The peak of IL-17 production was shown to coincide with the peak of IL-9 production both in splenocytes and DLN.

### In vivo Treatment of IFN-β Suppresses IL-9 Production in EAU

**Figure 2:**
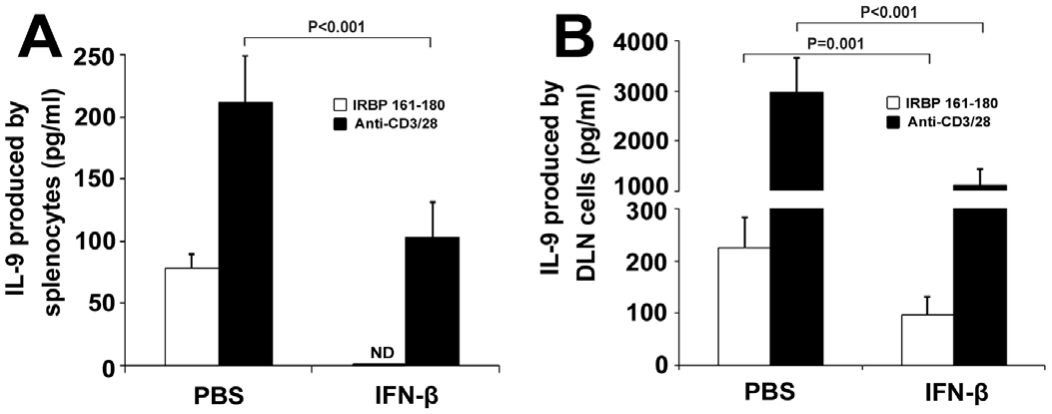
Effect of IFN-β treatment on IL-9 production. Splenocytes (A) and DLN cells (B) from the immunized mice following IFN-β or PBS treatment were activated with IRBP_161–180_ (20 µg/ml) or anti-CD3/CD28 (1 µg/ml) for 3 days. IL-9 was analyzed by ELISA. Data are representative of three independent experiments with at least five mice per group. ND: not detected.

After multiple administration of IFN-β to EAU mice, its effect on EAU activity and IL-9 production by stimulated splenocytes or DLN cells was assessed. As we reported previously, IFN-β significantly inhibited EAU activity during the inflammatory phase, as judged by both clinical and pathological evaluation [16]. In the present study, we found that the production of IL-9 by anti-CD3/CD28 or IRBP peptide stimulated splenocytes and DLN cells was significantly suppressed in IFN-β-treated mice as compared with the control PBS-treated mice (PBS-treated mice vs. IFN-β-treated mice, activated by anti-CD3/CD28∶211.8±38.0 pg/ml vs. 102.9±28.7 pg/ml in splenocytes; 2978.0±687.3 pg/ml vs. 1116.1±329.2 pg/ml in DLN, both P<0.001. Activated by IRBP_161–180_∶78.0±11.4 pg/ml vs. not detected in splenocytes; 225.9±57.6 pg/ml vs. 96.6±35.6 pg/ml in DLN, P = 0.001) (Figure 2).

### IFN-β Inhibits IL-9 Production in vitro

**Figure 3:**
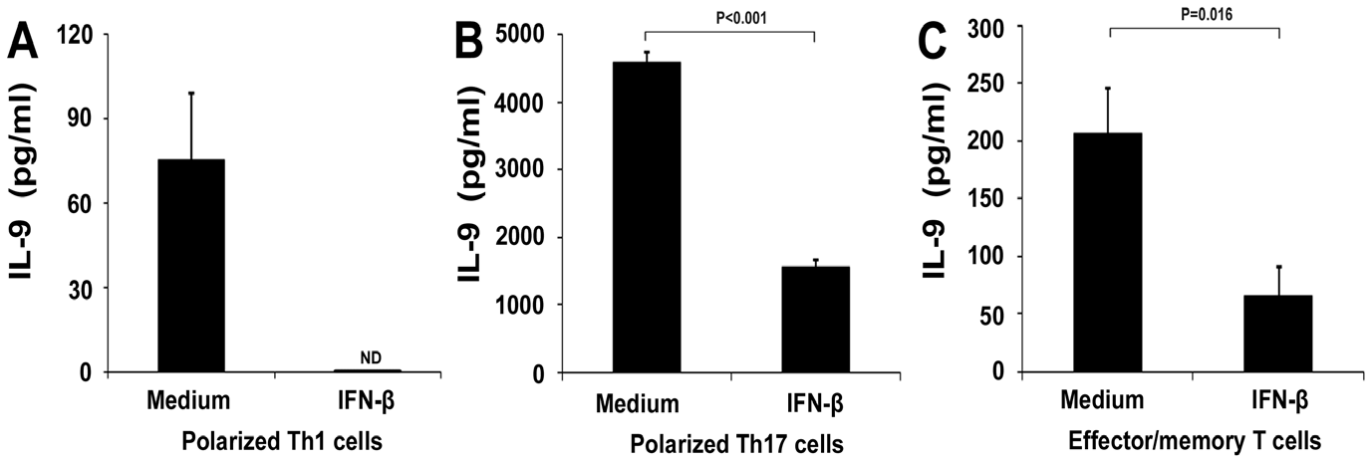
Effect of IFN-β on IL-9 production by polarized Th1, Th17 cells and effctor/memory T cells. Naïve T cells cultured with or without IFN-β in Th1-polarizing conditions (10 ng/ml IL-12) (A) or Th17-polarizing conditions (20 ng/ml IL-6, 5 ng/ml TGF-β1 and 10 ng/ml IL-23) (B) for 4 days. Effector/memory T cells were cultured with or without IFN-β for 3 days. IL-9 was analyzed by ELISA. Data are representative of three independent experiments.

Since Th1 and Th17 cells are implicated in the development of EAU, and IL-9 is also produced by these two Th subsets, we investigated whether IFN-β could influence the production of IL-9 by Th1 and Th17 cells. Naïve T cells were cultured under Th1 or Th17 polarizing conditions and IL-9 expression was investigated following anti-CD3/CD28 stimulation. We found that IL-9 production by Th17 cells was much higher than that by Th1 cells (4582.1±140.9 pg/ml vs. 75.5±23.6 pg/ml, P<0.001). Addition of IFN-β to the cultures markedly suppressed IL-9 production by both lymphocyte subsets. In the polarized Th1 cells, IFN-β treatment decreased the level of IL-9 below the detection limit (Figure 3A). In the polarized Th17 cells, IL-9 production decreased by more than 60% in the IFN-β treated group (4582.1 pg/ml to 1555.2 pg/ml, P<0.001) (Figure 3B).

IFN-β also suppressed IL-9 production by effector/memory T cells stimulated with anti-CD3/CD28 (206.7±60.6 pg/ml vs. 65.2±10.6 pg/ml, P = 0.016) (Figure 3C).

**Figure 4:**
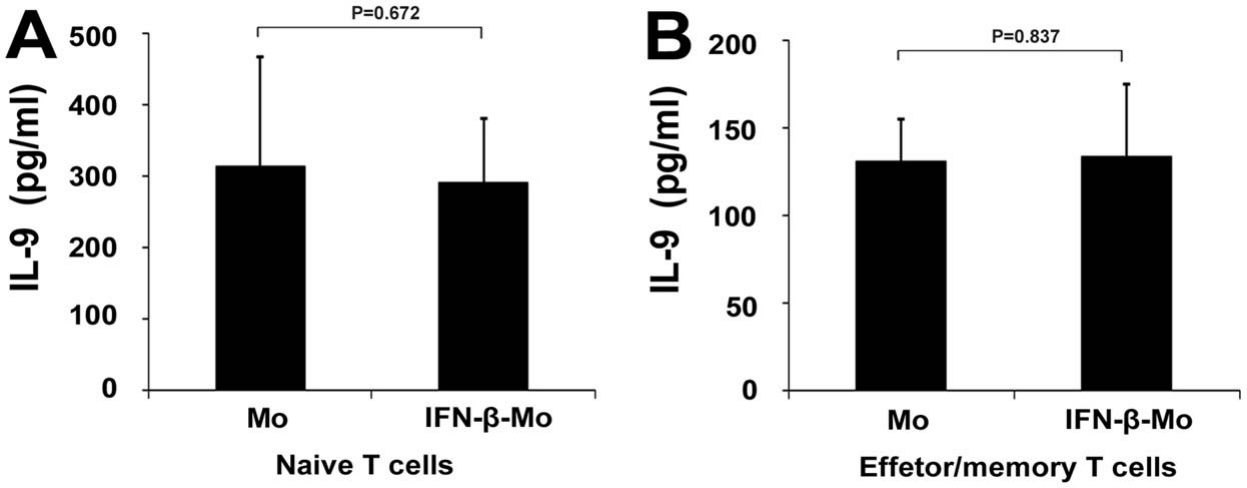
Effect of IFN-β-treated monocytes on the expression of IL-9. Naïve T cells (A) or effector/memory T cells (B) were cultured with monocyts (Mo) or IFN-β-treated monocytes (IFN-β-Mo) at a ratio of 5∶1 for 3 days. IL-9 levels were detected by ELISA. Data are representative of three independent experiments.

We previously found that IFN-β could inhibit IL-17 production by polarized Th17 cells indirectly through monocytes [16]. A further study was performed to test whether IFN-β-treated monocytes could also exert a suppressing effect on IL-9 production. However, the results showed that IFN-β-treated monocytes did not influence IL-9 production when cocultured with naïve T cells or with effector/memory T cells (Figure 4).

## Discussion

In this study we showed that production of IL-9 was upregulated during the active phase of EAU. IFN-β treatment suppressed EAU inflammatory activity and was associated with a significantly decreased expression of IL-9.

Until now only few groups have addressed the role of IL-9 in the pathogenesis of uveitis. IL-9 has been shown to enhance proliferation and IFN-γ production by an IRBP specific T cell line [19] and analysis of aqueous humor from uveitis patients reveals an increased IL-9 mRNA and protein level [20]–[21]. To our knowledge this is the first study investigating the role of IL-9 and IFN-β in EAU and supports the earlier studies suggesting a role for this cytokine in the pathogenesis of uveitis. A previous study by Tan et al. [22] reported that adoptive transfer of antigen specific Th9 cell lines was able to cause inflammation in recipient eyes expressing the antigen hen egg lysozyme. This intraocular inflammation model differs from ours with respect to the inciting antigen, but it does show a direct role for IL-9 producing cells in its pathogenesis. To prove a direct role for IL-9 in the development of IRBP induced EAU, further experiments using adoptive transfer, IL-9 blockade and IL-9 knockout animals are needed.

We studied the role of IL-9 and IFN-β during EAU by testing the cells obtained from the draining lymph nodes and spleens. These experiments revealed that IFN-β treatment markedly inhibited IRBP_161–180_-specific production of IL-9. Further experiments with purified lymphocyte subsets showed that IFN-β significantly inhibited IL-9 secretion by polarized Th1, Th17 cells and effector/memory T cells. In our previous study, we also demonstrated that IFN-β could significantly inhibit Th17 differentiation [16], which is in accordance with the prior observations by others [23]–[24]. In the present study, we observed that IL-9 production was much higher under Th17 polarization conditions and that IFN-β could significantly inhibit this secretion. However, the suppression on IL-9 production by IFN-β may not be specific, since the decreased level of IL-9 noticed here could also be due to an effect on Th17 differentiation leading to a lower amount of IL-9.

We further tested that whether IFN-β inhibited IL-9 production indirectly through monocytes. The results showed that IFN-β-treated monocytes did not influence IL-9 production when cocultured with naïve T cells or effector/memory T cells. Combined with the previously reported observation that IFN-β inhibited Th17 cells differentiation in vivo [25] and in vitro [23]–[24], [26], indicates that the amelioration of disease by IFN-β treatment may possibly not be due to the decreased level of IL-9 production by antigen specific cells but due to a decreased number of Th17 cells. Further studies are needed to clarify this issue.

When testing the DLN cells or splenocytes we did not observe a constitutive production of IL-9. The cells needed to be stimulated with anti-CD3/CD28 or the inciting IRBP peptide to generate IL-9 expression. As expected a higher response was observed when stimulating with anti-CD3/CD28 as compared to using IRBP peptide. Furthermore a higher IL-9 response was observed in DLN cells than in splenocytes. When using anti-CD3/CD28 as a second signal for T cell stimulation, there was no difference in IL-9 production in splenocytes between the inflammatory phase EAU mice, recovery phase EAU mice and the control mice. The explanation for the latter observation is not clear but may be due to a higher level of antigen specific cells in the DLN as compared to the spleen. On the other hand the unresponsiveness by splenocytes might be due to the local environment in the spleen during the induction of EAU. In our previous study [16], we found an increased expression of IFN-β in the inflammatory phase of EAU and the IFN-β was mainly produced by splenocytes but not by DLN cells. It is possible that the high endogenous splenic IFN-β may influence IL-9 production.

A study performed in humans showed that IFN-β pre-treated APC was able to inhibit MBP-specific T cell proliferation [27]. In our previous study we showed that IFN-β could directly reduce T cell proliferation, but we failed to find that IFN-β-treated monocytes could also reduce T cell proliferation [16]. Further reports showed that in IFN-β-treated Multiple Sclerosis patients, T cell proliferation was markedly reduced [28]–[29]. In view of these findings, we suspected that our treatment strategy of IFN-β from the very early phase of the disease alleviated EAU partially though directly inhibiting general T cell proliferation, not specifically decreasing the IL-9 producing cells. Whether the treatment strategy of IFN-β or IFN-β-treated antigen specific Th cells during the adaptive phase also suppressed inflammation needs further study.

Previous studies have shown that IL-9 plays a pro-inflammatory role during the development of autoimmune disease [14]–[15], [30]–[33]. Our findings during EAU support a pro-inflammatory role of IL-9, but recent studies have pointed out a dual role for this cytokine and have suggested an anti-inflammatory function depending on the phase of the immune response [34]. Initial studies in the experimental autoimmune encephalitis model showed that IL-9 neutralization or IL-9 knock out could attenuate disease [14]–[15], [32] but recent studies indicate that IL-9 may also play a role in Treg function whereby the outcome of the autoimmune disease may depend on the IL-9 mediated regulation of the Treg/Th17 balance [34]. Further studies using IL-9 neutralization or IL-9 knockout mice are needed to examine the exact role of IL-9 during the development of EAU and whether timing of IL-9 during the immune response may lead to a different outcome of the intraocular inflammatory response. This knowledge is necessary before a role of IL-9 in the treatment of uveitis can be envisaged.
